# Preliminary study on the anti-CO_2_ stress and growth ability of *hypsizygus marmoreus* mutant strain HY68

**DOI:** 10.1186/s12866-023-03050-1

**Published:** 2023-10-17

**Authors:** Fang Liu, Lin Ma, Weifeng Chen, Sifan Wang, Chuanzheng Wei, Chengpo Huang, Yimin Jiang, Song Wang, Hongyan Lin, Jian Chen, Gang Wang, Baogui Xie, Zongsheng Yuan

**Affiliations:** 1 College of Life Sciences, Fujian Agriculture and Forestry University, Fuzhou, Fujian 350002 China; 2 Future Technology Academy, Fujian Agriculture and Forestry University, Fuzhou, Fujian 350002 China; 3 Fujian Wanchen Biotechnology Group Stock Co., Ltd., Zhangzhou, Zhangpu, Fujian 363299 China; 4 Fuzhou Institute of Agricultural Sciences, Fuzhou, Fujian 350002 China; 5 Wetland College, Yancheng Teachers College, Yancheng, Jiangsu 224008 China; 6 College of Geography and Oceanography, Minjiang University, Fuzhou, Fujian 350108 China

**Keywords:** *Hypsizygus marmoreus*, Growth rate, β-glucosidase, Gene expression, Structure prediction

## Abstract

**Background:**

A high concentration of CO_2_ will stagnate the development of the newly formed primordia of *Hypsizygus marmoreus*, hinder the development of the mushroom cap, thereby inhibiting the normal differentiation of the fruiting body. Moreover, in the previous experiment, our research group obtained the mutant strain HY68 of *H. marmoreus*, which can maintain normal fruiting under the condition of high concentration of CO_2_. Our study aimed to evaluate the CO_2_ tolerance ability of the mutant strain HY68, in comparison with the starting strain HY61 and the control strain HY62. We analyzed the mycelial growth of these strains under various conditions, including different temperatures, pH levels, carbon sources, and nitrogen sources, and measured the activity of the cellulose enzyme. Additionally, we identified and predicted β-glucosidase-related genes in HY68 and analyzed their gene and protein structures.

**Results:**

Our results indicate that HY68 showed superior CO_2_ tolerance compared to the other strains tested, with an optimal growth temperature of 25 °C and pH of 7, and maltose and beef paste as the ideal carbon and nitrogen sources, respectively. Enzyme activity assays revealed a positive correlation between β-glucosidase activity and CO_2_ tolerance, with Gene14147 identified as the most closely related gene to this activity. Inbred strains of HY68 showed trait segregation for CO_2_ tolerance.

**Conclusions:**

Both HY68 and its self-bred offspring could tolerate CO_2_ stress. The fruiting period of the strains resistant to CO_2_ stress was shorter than that of the strains not tolerant to CO_2_ stress. The activity of β-GC and the ability to tolerate CO_2_ were more closely related to the growth efficiency of fruiting bodies. This study lays the foundation for understanding how CO_2_ regulates the growth of edible fungi, which is conducive to the innovation of edible fungus breeding methods. The application of the new strain HY68 is beneficial to the research of energy-saving production in factory cultivation.

*Hypsizygus marmoreus* is a species of Basidiomycota, Paramycota, Paramycota, and Dimycota, primarily cultivated in southern China [1], where it boasts a significant market potential due to its high annual output, delectable taste, and medicinal and nutritional properties [2–6]. As an aerobic heterotrophic fungus, the cultivation of *H. marmoreus* is divided into two stages: mycelial development and fruiting body growth [7]. These stages are influenced by several factors such as temperature, pH value, carbon and nitrogen sources, and CO_2_ concentration in the air [8]. Especially if the CO2 concentration in the air exceeds 1%, it will obviously inhibit the development of the fruiting body cap and affect the normal appearance of the fruiting body [8]. Moreover, producers currently grapple with the challenge of shortening the cultivation period, which stands at 120 days. Although the breeding of fine varieties offers an effective approach to these issues, these problems remain far from being fundamentally resolved [9], and research into the underlying molecular mechanisms is still scarce.

In the context of cultivating *H. marmoreus*, cellulose substances such as straw, corncobs, and other similar materials are added to the growth medium [10]. As the mycelium grows, it secretes a considerable number of extracellular enzymes [11], which effectively break down the cellulose and lignin present in the culture material into smaller molecular substances, such as glucose [12]. These substances are then easily absorbed and utilized by the mycelium, providing it with a sustainable and reliable carbon source [13]. Carboxymethyl cellulase, for example, is an enzyme that can break down cellulose to produce glucose and cellooligosaccharide [14]. Similarly, β-Glucosidase (EC3.2.1.21, β-GC) can hydrolyze oligosaccharides and disaccharide cellobiose into readily available glucose [15]. The content of corn cob in the cultivation material of *H. marmoreus* in this study was as high as 47%, which was the main source of cellulose, and makes cellulose account for an important proportion in the cultivation material. Therefore, the level of the enzyme activity of mycelia to degrade cellulose becomes the key to whether the strain of *H. marmoreus* can effectively utilize the culture substrate [16].

Due to *H. marmoreus’* long growth cycle, adequate ventilation is essential during the fruiting period to maintain CO_2_ concentration at approximately 0.3% [17, 18]. However, excessive ventilation may lead to the air-dried dehydration of the mushroom cap’s epidermal cells and result in scars [19], which can adversely affect the mushroom’s quality and sales. If strains that are tolerant to high levels of CO_2_ exist, ventilation can be reduced, which can save energy, including electricity, while also preventing scar formation. In previous experiments, our research group got multiple mutant strains of *H. marmoreus* through mutagenesis. One of these mutant strains, HY68, was found to be more tolerance to high concentration of CO_2_ during the fruiting period. As a result, this study aims to investigate HY68’s mycelial growth ability, focusing on its physiological and biochemical characteristics to explore the underlying mechanisms of CO_2_ tolerance.

## Materials and methods

### Tested strains and medium

The tested strains were provided by the Straits Research Institute of Fujian Agriculture and Forestry University:

HY61: starting strain, one of the commonly used strains in factory production,

HY68: mutagenic strain, the protoplasts of HY61 were obtained by EMS (ethyl methanesulfonate) mutagenesis.

HY62: external reference strain, one of the commonly used strains in factory production.

PDA medium: Potato 200 g, Glucose 20 g, Agar 20 g, Distilled water 1000 mL.

Basal medium: Peptone 8 g, Glucose 20 g, KH_2_PO_4_ 1 g, MgSO_4_ 0.5 g, VB1 10 mg, Agar 20 g, Distilled water 1000mL, pH 6.5.

Cultivation material formula: corn cob 47%, rice bran 29%, bran 7%, cottonseed hull 2%, soybean hull 5%, sugar beet meal 8%, shell powder 2%, water content 70%, pH value: 6.5.

### Mycelia growth experiment under high concentration of CO_2_

We used a sterilized hole puncher with a diameter of 6 mm to intercept the mycelial blocks of the activated strains to be tested and inoculated them in PDA medium. After 48 h of culture in a 25 °C incubator at a constant temperature, the culture dish was opened under sterile conditions and then placed in a sterile culture box. The culture box was then filled with carbon dioxide by the CO_2_ cylinder until the final concentration of CO_2_ in the air reached 4%, after which the box was sealed and placed in an incubator at 25 °C for cultivation. The CO_2_ concentration was checked every 2 days, and the CO_2_ in the culture box was supplemented to maintain a concentration of 4%. Three replicates were set up, and the mycelial growth was observed and recorded. When the radius of the fastest-growing colony reached 4/5 of the radius of the plate, the culture was stopped, and the diameters of all colonies were measured using the “cross” method [20, 21]. Each plate was randomly measured three times, and the average value was taken.

### Determination of mycelial growth ability under different conditions

Temperature experiment [22]: Sterile punchers with a diameter of 6 mm were utilized to evenly intercept activated mycelium blocks, which were then inoculated in PDA medium and placed in incubators at temperatures of 5 °C, 15 °C, 25 °C, and 35 °C, respectively, and culture them in the dark. Three replications were established for each temperature, and the growth of the mycelium was subsequently observed and recorded, with the colony diameter measured using the “cross” method. To ensure accuracy, each plate was randomly measured three times, and the average value was calculated.

pH experiment: use a sterile puncher (diameter 6 mm) to evenly intercept the activated mycelium blocks, inoculate them on PDA medium with different pH values, set the pH to 4, 5, 6, 7, 8, and 9 by sterile solution of 0.1 mol/L NaOH and 1% HCl. Each treatment was replicated 3 times. The plates were incubated in the dark at a temperature of 25 °C, and the growth of mycelium was observed and recorded using the same procedure as above [22].

Carbon source test: Taking the carbon content of 20 g glucose in the basal medium as the standard, replace 20 g of glucose in the basal medium with sucrose (18.2 g), maltose (19.0 g), soluble starch (18.2 g), lactose (19.0 g) with equal carbon content respectively, while maintaining all other components and keeping pH at 6.5. The mycelial culture and measurement methods were the same as above.

Nitrogen source test: Taking the nitrogen content of 8 g peptone in the basic medium as the standard. Urea (2.48 g), ammonium chloride (4.48 g), ammonium nitrate (3.36 g), yeast powder (12.44 g) with equal nitrogen content were used to replace 8 g peptone in the basic medium respectively, while maintaining all other components and keeping pH at 6.5. The mycelial culture and measurement methods were the same as above.

### Determination of extracellular enzyme activity in

#### Mycelium culture stage

**β-Glucosidase (EC3.2.1.21, β-GC)** [23–25]: shake culture bacteria in enzyme-producing medium (cellobiose 10 g, rice bran 3 g, bran 1 g, water 1 000 mL, pH value: 6.5) silk 4d. Take 0.5mL of the culture solution (crude enzyme solution) from which mycelium has been removed and use the Solebol β-glucosidase activity detection kit (BC2560) for enzyme activity detection. Under the condition of wavelength 400 nm, the increase rate of absorbance was measured to calculate the activity of β_GC. Definition of β-glucosidase activity unit: 1 µmol of p-nitrophenol per milliliter of crude enzyme solution per hour is defined as an enzyme activity unit (U/mL).

**Carboxymethyl cellulase** [25, 26]: The mycelia were cultured by shaking in the enzyme-producing medium (10 g sodium carboxymethylcellulose, 3 g rice bran, 1 g bran, 1 000 mL water, pH value: 6.5) for 4 d. Take 0.5mL culture solution (crude enzyme solution) from which mycelium has been removed, and use Solebo Cellulase Activity Detection Kit (BC2540) for enzyme activity detection. Take the enzyme solution that was inactivated by boiling for 15 min as a control. Measure the OD value at 520 nm. The glucose standard curve regression equation (y = 5.773x + 0.008, where y is the absorbance, x is the glucose concentration, and the unit is mg/mL) was used to determine the amount of glucose produced by the reaction. 3 replicates for each strain. Enzyme activity is defined as 1 µg of glucose catalyzed per minute in the reaction system per milliliter of crude enzyme solution is defined as an enzyme activity unit.

**Filter paper cellulase activity** [27]: The mycelia were cultured by shaking in the enzyme-producing medium (filter paper fiber 10 g, rice bran 3 g, bran 1 g, water 1000mL, pH value: 6.5) for 4d. Take 0.5mL culture solution (crude enzyme solution) from which mycelium has been removed, and use Solebo Cellulase Activity Detection Kit (BC2540) for enzyme activity detection, and take the enzyme solution that has been inactivated by boiling for 15 min as a control. Measure the OD value at 520 nm. 3 replicates for each strain. Standard curve and enzyme activity are defined as above.

#### Cultivation stage

**Fig. 1:**
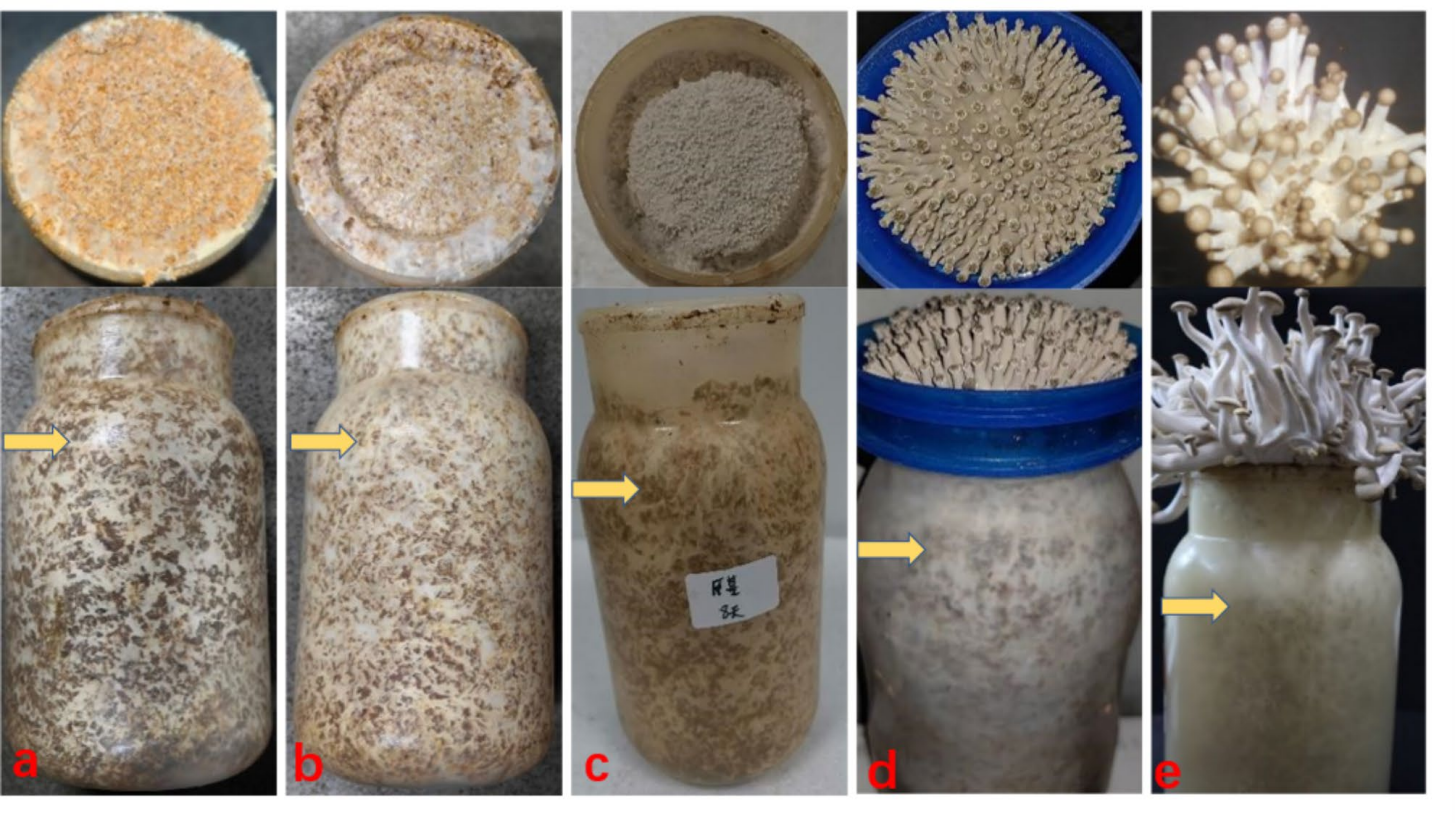
The different stages of HY68 cultivation and fruiting, and the collection of medium substrate samples. All we collected were the cultivation substrates indicated by the arrows. **a**: The samples were collected during the full mycelium period in the bottle (ZJMD). **b**: after the mycelium was post-ripened for 60 days (ZJHS. It takes about 60 days for the fermented substrate to produce mushrooms after the mycelium of the mushroom is full, otherwise the fruiting body output is extremely low). **c**: the period when the hyphae kink to produce the fruiting body primordium (ZJDT). **d**: the period when the fruiting body cap diameter is less than 3 mm (ZJ3G. At this stage, no basidioides and basidiospores were observed after repeated microscopic observation of the gills). **e**: the period when the fruiting body cap diameter is greater than 7 mm. (ZJ7G. At this period, a significant number of basidioides and basidiospores were observed through repeated microscopic observation of the gills)

**β-Glucosidase (EC3.2.1.21, β-GC)** [23–25]: To determine the activity of β-GC, substrates were collected at five different periods during the cultivation stage of HY68 based on the morphological changes observed (refer to Fig. 1). Each substrate sample (0.2 g) was ground in sterile conditions with 1 ml of buffer solution, followed by centrifugation to obtain the crude enzyme solution. The enzyme activity determination was the same as that of mycelium culture stage.

### Determination of β-GC-related gene expression

Previous studies [28] were consulted to search for complete protein and CDS sequences of β-GC genes [29, 30] in closely related species such as *Coprinus cinerea* through the NCBI website. Using HY61 as the reference genome, relevant genes and family information of β-GC were extracted through whole genome searches. The mycelium block of HY68 was inoculated into a cultivation bottle containing cultivation materials and sent to Fujian Wanchen Biotech Stock Co., Ltd. for cultivation and production. During the cultivation process, samples of the cultivation substrate were collected at five different stages (Fig. 1) and sent to Wuhan Feisha Biological Co., Ltd. to extract their RNA [31]. The expression of β-GC-related genes in the matrix at different cultivation periods was detected using fluorescence quantitative PCR, with the 18 S rRNA of HY68 serving as the internal reference gene. The relative expression of the target gene was calculated using the threshold comparison method [32].

### Prediction and analysis of β-GC-related genes

**Gene structure prediction (introns and exons)**: Submit the full sequence of β-GC-related genes to Augustus (http://bioinf.uni-greifswald.de/augustus/submission.php) to analyze introns and exons. **Gene transmembrane structure prediction**: the protein sequence of β-GC-related genes was submitted to TMHMM-2.0 (https://services.healthtech.dtu.dk/service.php?TMHMM-2.0) to obtain the transmembrane structure of the gene’s protein. **Gene signal peptide prediction**: Submit the protein sequence to the SignalP 6.0 server (https://services.healthtech.dtu.dk/service.php?SignalP) to locate the signal peptide(s) and identify signal peptide sites. **Gene protein structure prediction**: submit the protein sequence of β-GC-related genes to SWISS-MODEL (https://swissmodel.expasy.org/interactive) to perform a comprehensive three-dimensional structure analysis of the protein. These steps were taken to ensure the accuracy of the predictions and provide a comprehensive understanding of the gene structures.

### Industrial fruiting of HY68 self-bred strain

Buckle the 7 mm cap of HY68 in a sterile empty plate and keep it moist for 1 day at 25 °C to obtain spore prints. The spore prints were eluted with a small amount of sterile water, the resulting spore suspension was spread on a PDA plate. Many small colonies grow on the plate after culturing at 25 °C for 2–3 days, and the small colonies are picked out and cultured separately. Select the colonies without lock-like associations to be compiled as monospore strains, and pair the monospore strains in pairs on the PDA plate. After 7 days, observe and transfer out the strains with lock-like association in the hyphae (the strain was a bisporus strain that had successfully paired and belonged to the F1 self-bred strain). These inbred strains were subjected to 4% CO_2_ growth experiments (see Sect. 1.2.1 for methods). Inoculate the strains with normal hyphae growth under normal air and significant difference in mycelial growth under 4% CO_2_ into the cultivation bottle at the same time and carry out the fruiting experiment together (see Sect. 1.2.4 for the method).

## Results and analysis

### The growth of mycelia of each strain under 4% CO2 concentration

**Fig. 2:**
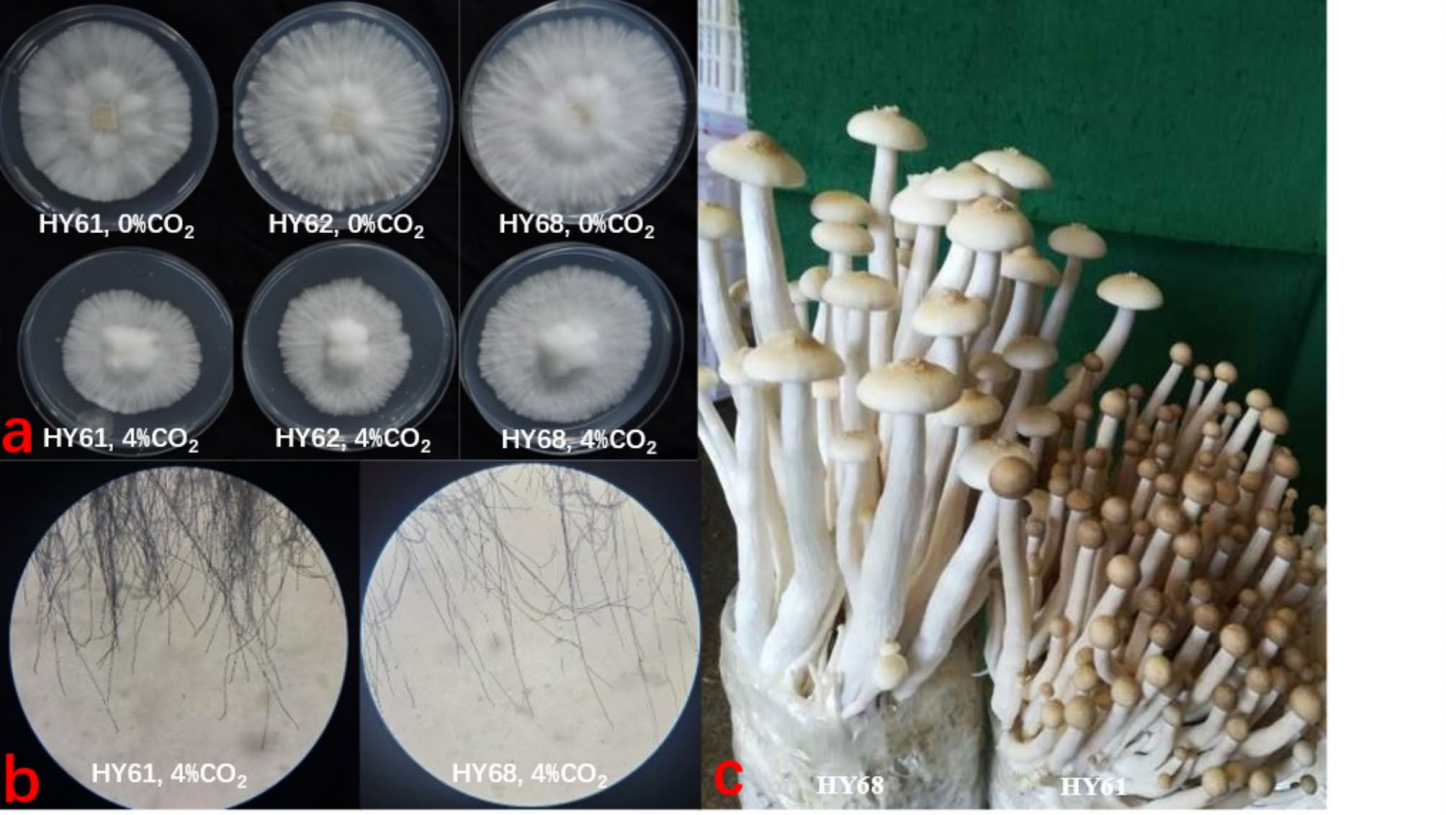
Displays the mycelial growth of each strain under a 4% CO_2_ concentration and at a temperature of 25℃. a: comparison of mycelial growth speed of each strain under normal air and 4% CO_2_ concentration. b: Under 4% CO_2_ concentration, the microscopic observation (100*) of the edge mycelial growth of HY61 (starting strain) and HY68 (mutant strain) colonies. c: Under normal cultivation conditions, the mushroom body growth of HY61 and HY68D on the 20th day after opening the cultivation bottle

The growth of mutant strain HY68 is lower as compared to 0% CO2, however it is not restricted as it shown for other two strains (Fig. 2). The growth rate under CO_2_ stress was: HY68 > HY62 > HY61. Moreover, microscopically, the marginal hyphae of the HY68 colony appeared looser than those of other strains at 4% CO_2_, with approximately 100 marginal hyphae visible in each 10*10x field of view. Conversely, the starting strain HY61 exhibited dense entanglement and gathering at the edge of the colony, impacting accurate counting. Both HY61 and HY68 were cultivated under uniform factory cultivation conditions (normal ventilation). On the 20th day after the cultivation bottle was opened (On the 115th day of the whole culture period), HY68 reached maturity and its fruiting body opened, while HY61 remained immature, with the fruiting body height just 2/3 that of HY68.

### Mycelia growth rate under different conditions

**Fig. 3:**
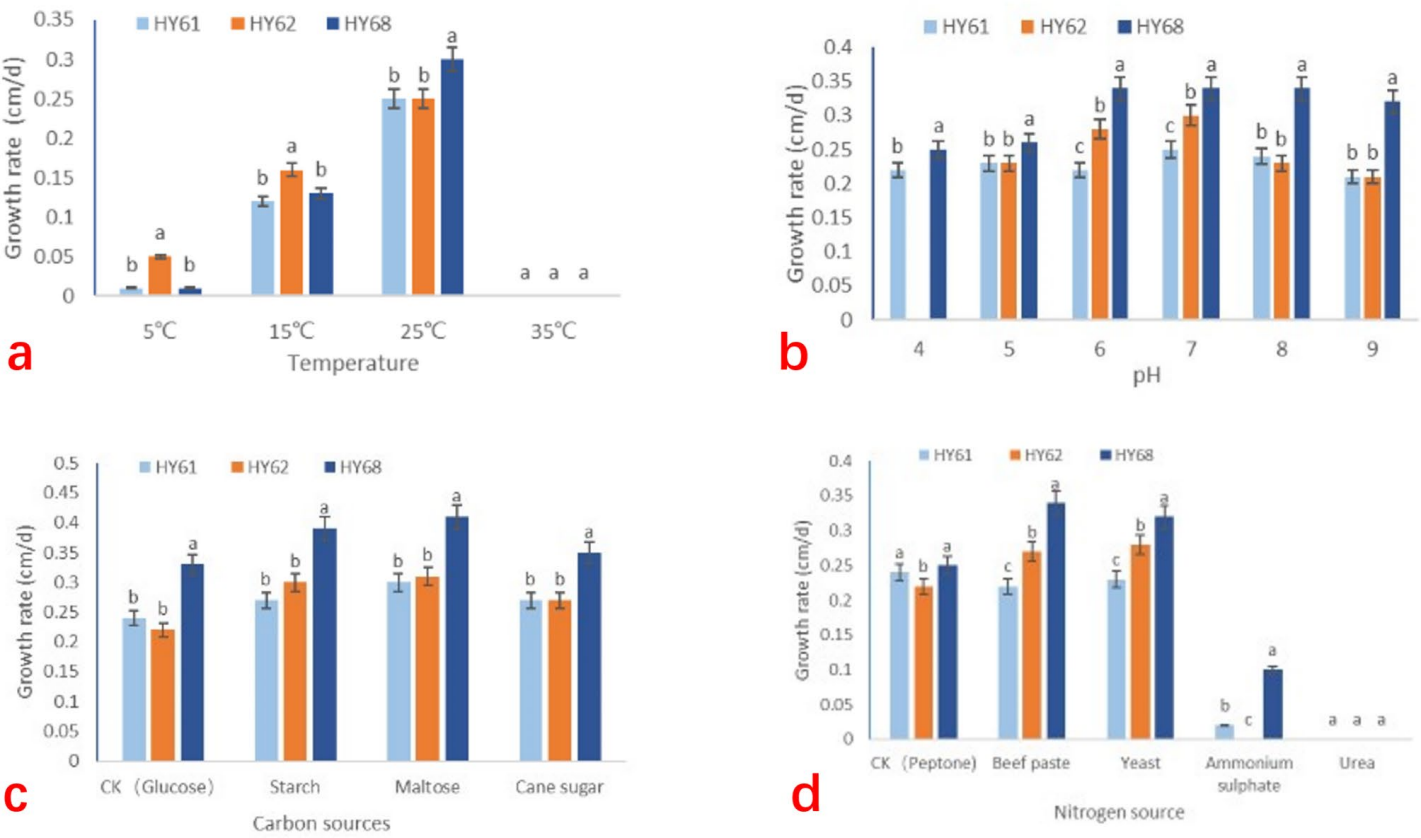
Various conditions affecting the growth rate of mycelia. **a**: The growth rate of mycelia under different temperature conditions. **b**: The effect of various pH values on mycelial growth rate. **c**: The growth rate of mycelia on the media with different carbon sources. **d**: The growth rate of mycelium on the medium of different nitrogen sources. (Different letters represent statistical differences (P < 0.05).)

Based on the data presented in Fig. 3a, it was evident that HY62 exhibits better growth on PDA medium at 5 and 15 °C, compared to HY68 and HY61. At 25 ℃, the growth rate of HY68 was faster than that of HY61 and HY62. However, at 35 °C, none of the tested strains could grow or stagnate. Generally, the growth rate of all three strains increased with temperature within the range of 5–25 ℃, with the fastest mycelial growth observed at 25 ℃. However, as the temperature continued to rise to 35 °C, the growth rate of mycelium fell to zero. Therefore, in summer, when the ambient temperature exceeds 35 °C, cooling measures must be taken to prevent stagnation of mycelium growth.

In Fig. 3b, it was evident that on PDA medium at 25 °C, HY61 and HY68 can grow within pH 4–9, while HY62 cannot grow at pH 4. Moreover, the mycelial growth rate of all strains was highest at pH 7, with HY68 exhibiting faster growth at different pH levels. The growth rate of other strains dropped rapidly under the pH range of 7–9, while HY68 sustained a high growth rate. Among the three strains, HY61 exhibited the smallest fluctuation in mycelium growth rate within the pH range of 4–9. Therefore, pH 7 can be considered the most optimal pH for mycelial growth of these three kinds of mushrooms. The control strain, HY62, exhibited no resistance to acid, whereas the mutant strain, HY68, demonstrated a certain degree of alkali resistance.

As revealed in Fig. 3c, mycelial growth rate was fastest under maltose carbon source with HY68 demonstrating the highest growth rate among the three strains. In contrast, glucose as a carbon source resulted in the slowest mycelial growth rate, with HY62 exhibiting the slowest growth among the three strains. Nevertheless, HY62 exhibited a higher average growth rate compared to HY61. Overall, growth rate under different carbon source conditions followed the order HY68 > HY62 > HY61, with HY68 exhibiting a significantly higher growth rate than the other two strains.

The Fig. 3d indicated that none of the three strains grew on urea as a nitrogen source. When the nitrogen source was ammonium sulfate, HY62 does not grow. On the other hand, HY68 exhibited the fastest growth rate on the medium supplemented with beef extract as the nitrogen source, which was considerably faster than the other two strains. Additionally, when the nitrogen source was yeast, beef extract or peptone, the overall average growth rate followed the order HY68 > HY62 > HY61.

### Determination of enzyme activity in mycelium culture stage

**Fig. 4:**
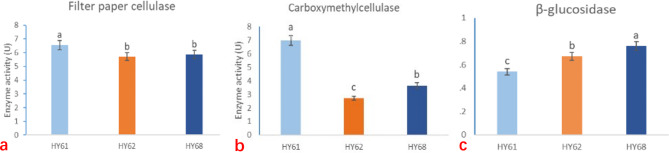
Enzyme activity during mycelium culture stage, **a**: activity of filter paper cellulase, **b**: activity of carboxymethyl cellulase **c**: activity of β-glucosidase (β- GC). (Different letters represent statistical differences (P < 0.05).)

To evaluate the cellulose-degrading ability of our strains, filter paper cellulase and carboxymethyl cellulase activities were measured and analyzed (Fig. 4). Based on the results, it was observed that the starting strain HY61 exhibited the highest activity for both enzymes, followed by HY68 during the mycelium culture stage. However, as these two enzymes were not able to completely break down cellulose into monosaccharides, the activity of β-GC was measured. Interestingly, the activity of β-GC in HY68 was found to be the highest among all the strains (Fig. 4c), while the activity in HY61 was the lowest. This trend was found to be consistent with the rate of mycelium growth, highlighting the role of β-GC in the enzymatic hydrolysis of available monosaccharides. Thus, further investigation was focused on this enzyme and its genes.

### Gene expression analysis of β-GC gene

**Fig. 5:**
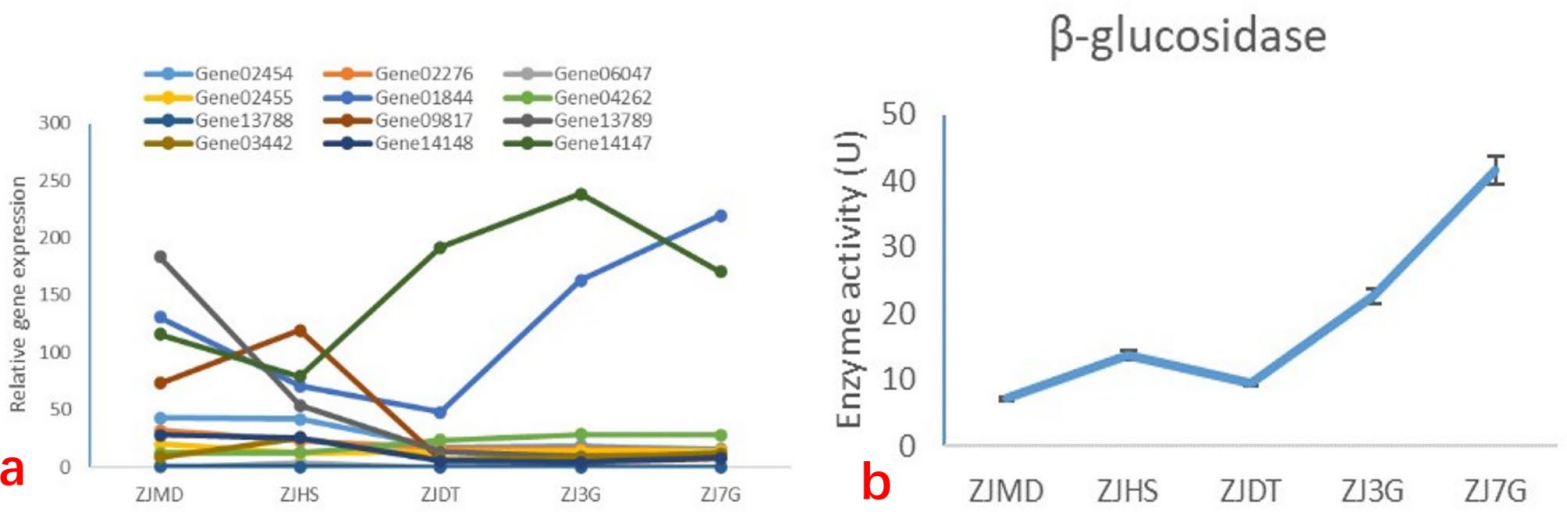
Illustrates the expression levels and enzyme activity of β-GC-related genes in HY68 during different stages of cultivation. **a**: Shows the β-GC-related gene expression at various cultivation stages. **b**: Presents the β-GC activity throughout HY68 cultivation. The cultivation stages include ZJMD (when the mycelium fills the bottle), ZJHS (the 60th day of post-ripening), ZJDT (when the hyphae start to produce the fruiting body primordium), ZJ3G (when the fruiting body cap diameter is less than 3 mm), and ZJ7G (when the fruiting body cap diameter is greater than 7 mm) (see Fig. 1 for detailed explanations)

By analyzing the genome-wide information of HY68, we identified 12 genes that are related to β-GC (Fig. 5). Following qPCR analysis, we discovered that four genes (Gene14147, Gene01844, Gene09817, and Gene13789) exhibited noticeable changes in expression, while the expression levels of the other genes were low during each stage (Fig. 5a). Gene09817 and Gene13789 demonstrated high expression only in the full bag and post-ripening periods, with the expression levels greatly reduced or remaining constant for the rest of the periods. Gene14147 exhibited high expression levels during the critical stage of fruiting body formation (primordia) and the 3 mm cap stage, with a slight decline observed during the 7 mm cap stage. In contrast, Gene01844 exhibited a significant increase in expression levels during the 3 mm cap stage and 7 mm cap stage. Similarly, the enzyme activity of β-GC in the five substrate samples increased significantly at the 3 mm cap stage and 7 mm cap stage, in line with the expression pattern of Gene01844. Although the cumulative impact of Gene14147’s high expression on the production of the β-GC enzyme cannot be excluded, we predicted the gene and protein structures of Gene14147 and Gene01844 (Fig. 5b).

Our experiments ended after the 7 mm cap stage, as we began harvesting HY68 at this stage, and the growing substrate began to shrink while allowing external air to enter the cultivation bottle, which may introduce other unwanted microorganisms. Therefore, we did not collect subsequent substrate samples after reaching the 7 mm cap stage.

### Structural analysis of β-GC-related genes

We performed gene structure prediction and predicted the transmembrane structure, signal peptide, and tertiary structure of the related gene proteins.

#### Gene structure prediction

**Fig. 6:**
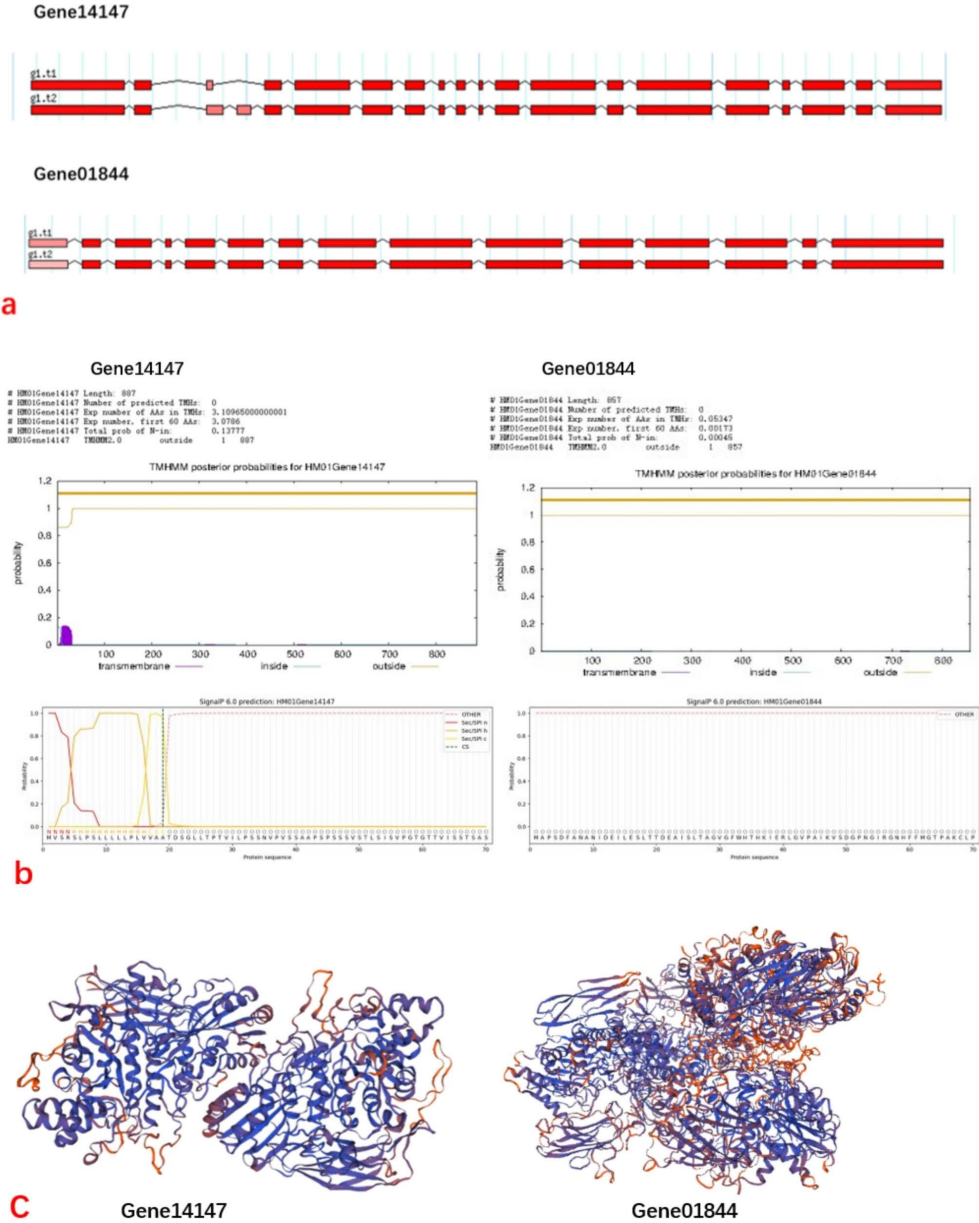
The prediction and analysis of β-GC-related genes and their proteins. **a**: Gene structure prediction of related genes. **b**: Prediction of transmembrane structure and signal peptide of related gene protein. **C**: Prediction of the tertiary structure of related gene proteins

Gene structure prediction revealed that Gene14147 comprises 19–20 exons and 18–19 introns, exhibiting two alternative splicing types. Similarly, Gene01844 contains 15 exons and 14 introns and has two alternative splicing types (Fig. 6a).

#### Prediction of transmembrane structure

Using TMHMM-2.0 to predict, the length of the Gene14147 protein sequence was 887, with an expected value of 3.08 amino acid residues in the transmembrane helix of the first 60 amino acids (Fig. 6b). The probability of the N-terminal located on the cytoplasmic side of the membrane was 0.14, indicating that the structure may be a transmembrane structure [33]. Conversely, the protein sequence length of Gene01844 was 857, and the prediction results show no transmembrane helix, with an expected value of 0.053 transmembrane helix amino acid residues. The probability of the N-terminal located on the cytoplasmic side of the membrane was 0.00045, ruling out the presence of a helical structure in the N-terminal region.

#### Signal peptide prediction

SignalP 6.0 tool was used to predict the protein’s signal peptide. Results exhibited the presence of Sec/SPI secretion signal peptide in the protein corresponding to Gene14147 gene, which was transported by Sec translocon and cleaved by signal peptidase I (Lep) at cleavage sites 18 and 19, with a probability of 0.976507 (Fig. 6b). However, no signal peptide was found in the protein corresponding to Gene01844 gene [34]. The signal peptide prediction outcomes are concurrent with the protein transmembrane structure prediction results.

#### Protein structure prediction

Protein structure prediction for Gene14147 gene displayed that the three-dimensional protein structure of Gene14147 was 88.06% sequence identity with β-GC’s 1 crystal structure from *Aspergillus aculeatus*. The protein’s three-dimensional structure for Gene01844 gene was only 43.41% sequence identity with Se-Met labeled β-GC’s crystal structure from *Kluyveromyces marxianus*. Based on the above data, it was indicated that Gene14147 may have a more significant effect on glucosidase activity than Gene01844, but the possibility of a synergistic effect of these two genes cannot be ignored (Fig. 6c).

### Growth difference of HY68 inbreds

**Fig. 7:**
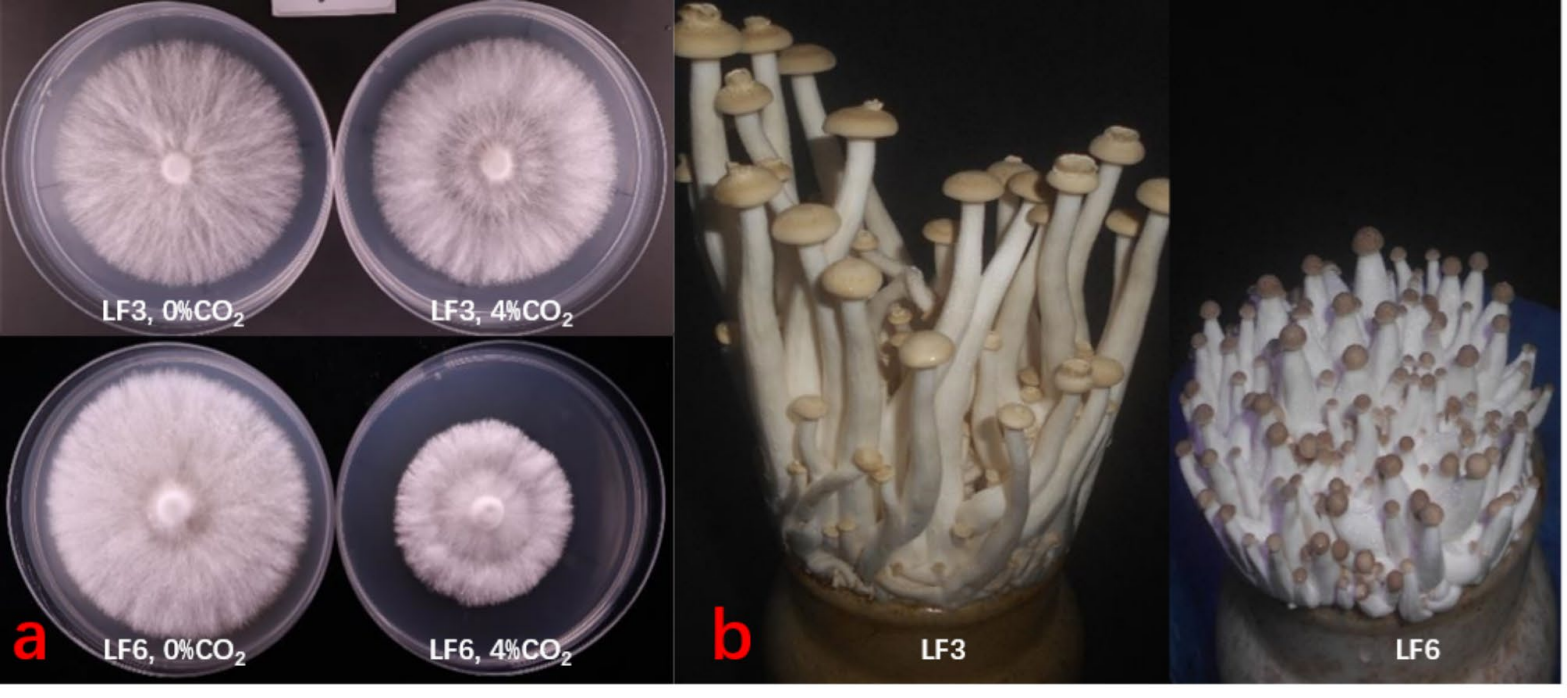
Growth differences of HY68 selfed strains. **a**: Shows the difference in the growth of mycelia on the plate PDA medium under CO_2_ stress. **b**: Shows the fruiting differences of the HY68 self-bred strain LF3 and LF6 under normal factory conditions, specifically on the 20th day after opening the cultivation bottle

Through monospore hybridization, we obtained many HY68 inbreds. In 4% CO2, these inbreds showed segregation of CO_2_ traits. For example: In selfing strains, LF3 was tolerant to 4% CO_2_, and LF6 was not tolerant to 4% CO_2_, but their growth rates are basically the same in normal air. In the case of 4% CO_2_, the mycelial growth rate of LF3 plate was 1.77 times that of LF6 (Fig. 7a). The selfing strains are cultivated under uniform factory cultivation conditions (normal ventilation), but the generation time of primordia and the time of mushroom formation are different. Compared to LF6, the primordium formation time of LF3 was 2 days earlier and its mushroom maturity time was 3–4 days earlier. On the 20th day after initiating cultivation, LF3 was close to maturity while LF6 showed limited signs of fruiting, LF6 fruiting bodies are less than half the height of LF3 (Fig. 7b).

## Discuss

### Correlation between the growth of *H. Marmoreus* and CO_2_ stress

In recent years, it has been found that CO_2_ is related to the stability of *Candida albicans* hyphae-specific transcription factor Ume6 and hyphal elongation [35]. The cell membrane of *Saccharomyces cerevisiae* was found to be a possible target in response to CO_2_ stress, causing cell growth inhibition [36]. People always think that high concentration of CO_2_ can inhibit or even poison the growth of edible fungi [37]. During the growth process, ventilators must be used to continuously inject fresh air into the mushroom house, otherwise the air conditioning in the mushroom house will not only affect the growth of mushrooms but also lead to disease. But, in the previous mutagenesis process, we accidentally found that the mutant strain HY68 can produce mushrooms normally under high concentration of CO_2_. So, a series of studies have thus been carried out. Interestingly, HY68, which was mutated from HY61, not only grew the fastest, but also tolerated various conditions more than the other two strains. For example, HY68 has a certain ability of alkali resistance, and the control strain HY62 was neither acid nor ammonium sulphate tolerant, while HY61 has the slowest growth rate.And in the recent fruiting experiment of HY68 inbreds, it was found that under 4% CO_2_ stress, the strains with fast mycelial growth rate also had faster mushroom body formation than the strains that did not tolerate 4% CO_2_.

### Kinetics of enzyme activity

The proportion of cellulose material in the cultivation material of *H. marmoreus* was the highest (corncob 47%, rice bran 29%). It was generally believed that the cellulose degradation ability was positively correlated with the fruiting body yield and biological efficiency of edible fungi [38]. Therefore, carboxymethyl cellulase, filter paper cellulase and β-GC, which can represent cellulase activity, were selected in this study to explore the internal reason of HY68 tolerance to CO_2_. However, in this study we found that the activities of carboxymethyl cellulase and filter paper cellulase were not related to the growth rate of mycelia. HY61 performed the best in the activities of carboxymethyl cellulase and filter paper cellulase, but its plate mycelial growth rate was not only the worst in different carbon source media, but also the slowest overall growth rate. The law of β-glucosidase (β-GC) activity was HY68 > HY62 > HY61, just in line with the law of mycelium growth rate of the three strains, and also consistent with the growth rate of mycelia in 4% concentration of CO_2_. β-GC is responsible for the further hydrolysis of cellotriose and cellulose oligosaccharides to generate directly available glucose, which is also the last key step in cellulose degradation. However, the proportion of β-GC in cellulase is very low, less than 1%. Our research found that the activity of β-GC in the cultivation medium increased rapidly during the period of fruiting body formation, which made β-GC one of the bottlenecks for the growth and energy supply of fruiting bodies. In the cultivation experiment, it was found that the fruiting body formation period of the strains that were not tolerant to CO_2_ stress and had low β-GC activity was longer than that of the strains tolerant to CO_2_ stress. Therefore, we believe that the cellulose degradation ability was positively correlated with the fruiting body yield and biological efficiency of edible fungi, which was not necessarily correct. The activity of β-GC and the ability to tolerate CO_2_ were more closely related to the growth efficiency of fruiting bodies.

### Analysis of β-glucosidase related genes

Based on the correlation between β-GC activity and CO_2_ tolerance and the growth efficiency of fruiting bodies, we further explored the changes of β-GC activity during cultivation and its related genes. We found that the genes whose gene expression levels changed relatively close to the changes of β-GC activity during cultivation were Gene14147 and Gene01844. The expression change of Gene01844 was consistent with the change of β-GC enzyme activity, it was predicted that there was neither transmembrane helical structure nor signal peptide at the N-terminus of the protein. Although the expression change of Gene14147 was not consistent with the change of β-GC enzyme activity, it was predicted that there was transmembrane helical structure and signal peptide at the N-terminus of the protein. However, the amount of gene expression can only explain the number of synthesized polypeptide chains [39], and complex modification and folding are required from polypeptide chains to active enzymes [40], so the amount of gene expression cannot fully represent the effective activity of extracellular enzymes [41]. Therefore, the expression change of Gene01844 was consistent with the change of β-GC enzyme activity but not necessarily related to its activity. The Gene14147 gene was predicted to have a transmembrane helical structure and the existence of the standard secretion signal peptide Sec/SPI at the N-terminal of the protein, it suggested that the protein of Gene14147 may function in the cytoplasm, which can guide its transfer to the secretory pathway and become an effective extracellular enzyme. And in terms of enzyme structure, it was highly related to the identified β-GC of Aspergillus aculeatus. Therefore, the correlation between Gene14147 and the extracellular enzyme activity of β-GC was higher than that of Gene01844, but there may also be a synergistic effect between Gene14147 and Gene01844.

In this study, we found that the activity of β-GC enzyme in the primordium period was the lowest, but it seemed to be an inflection point in the whole growth cycle. After this period, the expression levels of β-GC and its related genes Gene14147 and Gene01844 were greatly increased. It was speculated that from primordia to mushroom body formation, mycelium undergoes kinks and undergoes rapid structural differentiation and firmness, all of which require a lot of energy support [42], so the formation of primordia was a critical period for the start of numerous monosaccharides to be utilized [43]. During this period, if the expression of β-GC gene cannot be improved, it was likely to affect the production of enzymes and then affect the formation of fruiting bodies and the establishment of reproductive growth.

## Conclusion

Both HY68 and its self-bred offspring have the ability to tolerate 4% CO_2_. The fruiting period of the strains resistant to high concentration CO_2_ was shorter than that of the strains not tolerant to high concentration CO_2_. The activity of β-GC and the ability to tolerate CO_2_ were more closely related to the growth efficiency of fruiting bodies. In the later stage, we will explore the feasibility study of using CO_2_ to select strains and investigate the relationship between CO_2_ stress and β-glucosidase activity, and further verification of the functions of β-GC-related genes.

## Data Availability

The datasets generated during the current study are not publicly available due Lin Ma and Weifeng Chen are studying master and undergraduate but are available from the corresponding author on reasonable request. The corresponding author is Fang Liu, email: fjliufang@163.com.
